# Association of Health Behaviors with Mental Health Problems in More than 7000 Adolescents during COVID-19

**DOI:** 10.3390/ijerph19159072

**Published:** 2022-07-25

**Authors:** Elke Humer, Thomas Probst, Jolana Wagner-Skacel, Christoph Pieh

**Affiliations:** 1 Department for Psychosomatic Medicine and Psychotherapy, University for Continuing Education Krems, 3500 Krems, Austria; thomas.probst@donau-uni.ac.at (T.P.); christoph.pieh@donau-uni.ac.at (C.P.); 2 Department of Medical Psychology and Psychotherapy, Medical University of Graz, 8036 Graz, Austria; jolana.wagner-skacel@medunigraz.at

**Keywords:** adolescents, physical activity, smartphone usage, depression, anxiety, insomnia, stress, COVID-19, health behaviors

## Abstract

Previous studies show detrimental effects of the COVID-19 pandemic and associated lockdowns on the lives of adolescents. Adolescents have experienced disruption in their daily routines, including changes in health behaviors such as an increased sedentary behavior and increased smartphone usage. The aim of this study was to assess the association of health behaviors with mental health problems in Austrian adolescents during the pandemic. Five cross-sectional surveys (February 2021 to May 2022) were performed during the pandemic assessing physical activity, smartphone usage, depressive symptoms (PHQ-9), anxiety symptoms (GAD-7), sleep quality (ISI-7), and stress (PSS-10). In total, *N* = 7201 adolescents (age: 14–20 years ((MW±SD): 16.63 ± 1.49 years); 70.2% female, 18.8% migration background) participated. A strong increase in mobile phone usage as well as a decrease in physical activity as compared to pre-pandemic data were observed (*p* < 0.001). Compared to the lowest smartphone user group (<1 h/d), the adjusted odds ratios (aOR) for all investigated mental health symptoms increased with increasing smartphone usage up to 3.2–6.8 in high-utilizers (>8 h/d). The aORs for depressive, anxiety, insomnia, and stress symptoms decreased in physically active compared to inactive adolescents. Results highlight the need for measures to promote responsible smartphone usage as well as to increase physical activity, so as to promote mental health in adolescence.

## 1. Introduction

The COVID-19 pandemic and associated lockdowns have a major impact on mental health, especially in young people [1,2,3]. There are undoubtedly many reasons for this, like social isolation, disrupted education, diminished job opportunities [4], less use of offers of help [5] or changed health behaviors, such as an increase in smartphone usage or a decrease in physical activity [6]. This is particularly relevant as physical activity not only exerts antidepressant effects [7], but also reduces the incidence of depression over the course of a lifetime [8].

This study assessed the association of health behaviors with mental health problems at five timepoints during the pandemic in Austrian adolescents and compared the results to pre-pandemic epidemiological data.

## 2. Materials and Methods

In total, *N* = 7201 adolescents (age: 14–20 years ((MW ± SD): 16.63 ± 1.49 years); 70.2% female, 18.8% migration background) participated in five cross-sectional online surveys between February 2021 and May 2022 (study sample see Table 1).

Mental health symptoms (depressive symptoms (PHQ-9 [9]), anxiety symptoms (GAD-7 [10]), sleep quality (ISI [11]), and stress (PSS-10 [12])) were assessed and analyzed for their association with smartphone usage (hours per day) and physical activity (days of at least 1 h physical activity per week).

Mental health indicators (depressive symptoms, anxiety symptoms, insomnia symptoms, and stress) were dichotomized (not clinically relevant; clinically relevant) according to established cut-offs. For the PHQ-9, a cut-off point ≥10 was used in participants aged 18 or older [13] to define clinically relevant depressive symptoms, whereas a cut-off ≥11 was used for adolescents aged between 14 and 17 [14]. For anxiety symptoms, cut-offs ≥11 in 14- to 17-year-old adolescents and ≥10 in 18- to 20-year-old adolescents on the GAD-7 scale were used [15,16]. ISI-scores ≥15 were considered to indicate clinically relevant insomnia [11]. PSS-10 scores ≥14 were categorized as moderate to high stress levels [12].

In all surveys, smartphone usage was assessed with the following single item question: “In a typical day, how much time do you spend—sitting or lying down—on your smartphone?”, with the following answer options: less than 1 h/d, 1 to 2 h/d, 3 to 4 h/d, 5 to 6 h/d, 7 to 8 h/d, more than 8 h/d. In all surveys, physical activity was assessed by a single item question asking, “On how many of the last 7 days were you physically active for at least 60 min?”.

Using SPSS version 26 (IBM Corp, Armonk, NY, USA), Chi-squared tests were conducted to analyze changes in health behaviors compared to pre-pandemic data [17] and to analyze their association with mental health indicators. Multivariable logistic regressions were applied to adjust the data for age, gender, migration background, school type, time, region, smartphone usage (6 categories), and physical activity (8 categories). Adjusted odds ratios (OR) and their 95% confidence intervals (CIs) were estimated to assess statistical uncertainty (*p*-values < 0.05 were considered statistically significant; 2-sided tests).

## 3. Results

### 3.1. Association of Smartphone Usage with Mental Health Problems

Compared to the lowest smartphone user group (<1h/d), the adjusted odds ratios (aOR) for depressive symptoms increased with increasing smartphone usage to 1.98 (1.28–3.06) (3–4 h/d), 3.30 (2.13–5.11) (5–6 h/d), 4.96 (3.14–7.83) (7–8 h/d), and 6.79 (4.28–10.78) (>8 h/d) (Table 2, *p* < 0.01). High utilizers (>8 h/d) were also more likely to experience clinically relevant anxiety, insomnia, or stress symptoms (aORs 3.23–5.75) compared to those using the smartphone less than 1 h/d (Table 2, *p* < 0.001).

The prevalence of all investigated mental problems increased with increasing time using the smartphone (*p* < 0.001; Table 3).

### 3.2. Association of Physical Activity with Mental Health Problems

The aORs for depressive symptoms decreased in physically active compared to inactive adolescents to 0.74 (0.60–0.91) (1 day physical activity), 0.59 (0.49–0.72) (2 days), 0.51 (0.41–0.62) (3 days), 0.44 (0.36–0.55) (4 days), 0.38 (0.30–0.48) (5 days), 0.29 (0.22–0.38) (6 days), and 0.35 (0.28–0.44) (7 days) (Table 4, *p* < 0.01). Being physically active daily decreased the odds for anxiety, insomnia, and stress symptoms (aORs 0.37–0.66) compared to being physically inactive (Table 4, *p* < 0.01).

Physically inactive participants had the highest prevalences for depressive, anxiety, insomnia, and stress symptoms (*p* < 0.001; Table 5).

### 3.3. Changes in Health Behaviors during COVID-19 Compared to Pre-Pandemic Times

Compared to pre-pandemic Austrian data from 2018 [17], smartphone usage increased during the pandemic and physical activity decreased (Table 6): 8.4% of young people did not exercise in 2018, during the pandemic it was 14.7%; high smartphone users (>8h/d) increased from 3.3% to 12.2% in the same period (all *p*-values < 0.001).

## 4. Discussion and Conclusions

The results of this study underline once again the association of smartphone usage and physical activity with mental health problems. All assessed mental health indicators occur more frequently in the high smartphone utilizer group and less often in physically active adolescents. This is of particular concern given the increase in smartphone use coupled with a decline in physical activity compared to pre-pandemic levels. However, for adolescents today—especially in times of the COVID-19 pandemic—online relationships may be the norm to share information and interact with peers [18]. Adolescents might also benefit from online access to productive mental health information, such as online interventions for mental health or low barriers to resources such as crisis interventions [19]. Smartphone applications might also be used to detect unhealthy habits by tracking health and health-related behaviors, such as exercise, sleep, diet, and stress among others [20]. Thus, smartphone applications also pose potential to facilitate improvements to lifestyle [21].

Appropriate approaches are needed to foster responsible smartphone usage to mitigate potential harms from excessive mobile phone usage. The observed associations between physical activity and mental health in adolescents further underscore the need to promote physical activity to prevent or intervene on mental health problems in adolescence. 

When interpreting the data, several limitations have to be considered. It was not possible to consider a potential participant overlap, as no personal data to identify adolescents were collected to ensure strictly anonymous data collection. A further drawback is the non-representative gender distribution, with an overrepresentation of female adolescents. Moreover, prevalences of mental health problems were estimated based on self-rating instruments and not validated by clinical assessments. Also, the time spent on the smartphone was not further differentiated (e.g., time spent for academic learning, time spent on social media platforms etc.). Finally, we cannot exclude a self-selection bias toward higher participation of adolescents with higher mental health burden attributable to the online nature of the study.

## Figures and Tables

**Table 1:** Study sample characteristics (*N* = 7201).

Variable	*N*	%
Age		
14	595	8.3
15	1181	16.4
16	1569	21.8
17	1785	24.8
18	1345	18.7
19	510	7.1
20	216	3.0
Gender		
Female	5056	70.2
Male	1994	27.7
Diverse	151	2.1
Migration background		
No	5722	81.2
Yes	1323	18.8
School type		
College for Higher Vocational Education	2820	39.2
Academic Secondary School	2744	38.1
Vocational Education/Apprenticeship	1527	21.2
Others	110	1.5
Time		
February 2021	3052	42.4
March–May 2021	1261	17.5
June–July 2021	720	10.0
September–November 2021	1505	20.9
April–May 2022	663	9.2
Region		
North-East (Vienna, Lower Austria, Upper Austria)	4322	60.0
South-East (Carinthia, Styria, Burgenland)	1599	22.2
West (Tyrol, Salzburg, Vorarlberg)	1280	17.8

Migration background was defined as whether both parents were born abroad (second-generation immigrants) or adolescents themselves were born abroad (first-generation immigrants). Numbers do not sum up to *N* = 7201 as information on migration background was not provided by all adolescents. Diverse indicates adolescents whose gender identity or gender expression does not conform to socially-defined male or female gender norms.

**Table 2:** Adjusted odds ratios and their 95% confidence intervals for different smartphone usage time categories vs. <1 h smartphone usage per day.

	Smartphone Usage (h/d) vs. <1 h/d				
	1–2 h/d	3–4 h/d	5–6 h/d	7–8 h/d	>8 h/d
Depressive symptoms	1.42	1.98	3.30	4.96	6.79
	(0.91–2.22)	(1.28–3.06)	(2.13–5.11)	(3.14–7.83)	(4.28–10.78)
Anxiety symptoms	1.22	1.42	2.05	2.50	3.96
	(0.78–1.92)	(0.92–2.20)	(1.32–3.19)	(1.59–3.93)	(2.51–6.24)
Insomnia symptoms	1.03	1.34	2.10	2.32	3.23
	(0.60–1.79)	(0.79–2.28)	(1.23–3.57)	(1.35–4.00)	(1.88–5.56)
Moderate or high stress	1.24	1.93	3.20	4.86	5.75
	(0.80–1.95)	(1.25–2.99)	(2.03–5.04)	(2.89–8.18)	(3.35–9.89)

Mental health indicators (depressive symptoms (PHQ-9 [9]), anxiety symptoms (GAD-7 [10]), insomnia symptoms (ISI [11]), and stress (PSS-10 [12])) were dichotomized (0 = not clinically relevant, 1 = clinically relevant) according to established cut-offs. As not all data were provided by all participating adolescents, total numbers of adolescents included in the analyses were: *n* = 6703 for depressive symptoms, *n* = 6771 for anxiety symptoms, *n* = 6934 for insomnia symptoms, and *n* = 6817 for stress symptoms.

**Table 3:** Proportion of participants exceeding the cut-off scores for moderate depressive, anxiety, insomnia, and stress symptoms by smartphone usage.

Variable	Smartphone Usage						*p*-Value
	<1 h/d	1–2 h/d	3–4 h/d	5–6 h/d	7–8 h/d	>8 h/d	
Depressive symptoms	28.2%	37.0%	46.8%	61.3%	71.8%	78.3%	<0.001
Anxiety symptoms	26.5%	31.9%	37.1%	48.3%	54.8%	66.1%	<0.001
Insomnia symptoms	14.3%	15.1%	19.3%	28.1%	30.9%	39.7%	<0.001
Moderate or high stress	68.4%	74.9%	83.6%	90.7%	94.4%	95.5%	<0.001

Mental health indicators (depressive symptoms (PHQ-9 [9]), anxiety symptoms (GAD-7 [10]), insomnia symptoms (ISI [11]), and stress (PSS-10 [12])) were dichotomized (0 = not clinically relevant, 1 = clinically relevant) according to established cut-offs. As not all data were provided by all participating adolescents, total numbers of adolescents included in the analyses were: *n* = 6703 for depressive symptoms, *n* = 6771 for anxiety symptoms, *n* = 6934 for insomnia symptoms, and *n* = 6817 for stress symptoms.

**Table 4:** Adjusted odds ratios and their 95% confidence intervals for different categories of physically activity vs. physically inactivity.

	Physically Active Days per Week vs. 0 Physically Active Days						
	1 Day	2 Days	3 Days	4 Days	5 Days	6 Days	7 Days
Depressive	0.74	0.59	0.51	0.44	0.38	0.29	0.35
symptoms	(0.60–0.91)	(0.49–0.72)	(0.41–0.62)	(0.36–0.55)	(0.30–0.48)	(0.22–0.38)	(0.28–0.44)
Anxiety	0.88	0.73	0.61	0.55	0.51	0.43	0.46
symptoms	(0.74–1.06)	(0.61–0.88)	(0.51–0.73)	(0.45–0.68)	(0.41–0.64)	(0.33–0.56)	(0.37–0.58)
Insomnia	0.82	0.72	0.67	0.64	0.60	0.47	0.66
symptoms	(0.68–1.00)	(0.60–0.88)	(0.55–0.81)	(0.51–0.80)	(0.47–0.77)	(0.35–0.64)	(0.52–0.85)
Moderate or high	1.02	0.77	0.60	0.51	0.43	0.35	0.37
stress	(0.73–1.44)	(0.56–1.05)	(0.44–0.82)	(0.37–0.71)	(0.32–0.60)	(0.25–0.50)	(0.27–0.50)

Mental health indicators (depressive symptoms (PHQ-9 [9]), anxiety symptoms (GAD-7 [10]), insomnia symptoms (ISI [11]), and stress (PSS-10 [12])) were dichotomized (0 = not clinically relevant, 1 = clinically relevant) according to established cut-offs. As not all data were provided by all participating adolescents, total numbers of adolescents included in the analyses were: *n* = 6703 for depressive symptoms, *n* = 6771 for anxiety symptoms, *n* = 6934 for insomnia symptoms, and *n* = 6817 for stress symptoms.

**Table 5:** Proportion of participants exceeding the cut-off scores for moderate depressive, anxiety, insomnia, and stress symptoms by physical activity.

		Physically Active Days per Week							*p*-Value
	0 Days	1 Day	2 Days	3 Days	4 Days	5 Days	6 Days	7 Days	
Depressive symptoms	71.6%	66.5%	59.1%	54.8%	48.7%	42.6%	37.7%	37.5%	<0.001
Anxiety symptoms	56.9%	54.8%	48.0%	43.2%	38.4%	34.5%	31.6%	29.8%	<0.001
Insomnia symptoms	34.1%	29.2%	25.3%	23.1%	20.9%	19.0%	16.0%	19.7%	<0.001
Moderate or high stress	92.5%	92.8%	89.9%	87.6%	83.8%	80.0%	77.7%	74.4%	<0.001

Mental health indicators (depressive symptoms (PHQ-9 [9]), anxiety symptoms (GAD-7 [10]), insomnia symptoms (ISI [11]), and stress (PSS-10 [12])) were dichotomized (0 = not clinically relevant, 1 = clinically relevant) according to established cut-offs. As not all data were provided by all participating adolescents, total numbers of adolescents included in the analyses were: *n* = 6703 for depressive symptoms, *n* = 6771 for anxiety symptoms, *n* = 6934 for insomnia symptoms, and *n* = 6817 for stress symptoms.

**Table 6:** Smartphone usage and physical activity in adolescents before the COVID-19 pandemic and during the COVID-19 pandemic.

Variable		Smartphone Usage							*p*-Value
		<1 h/d	1–2 h/d	3–4 h/d	5–6 h/d	7–8 h/d	>8 h/d		
Pre-pandemic		3.6%	25.7%	41.9%	19.3%	6.2%	3.3%		<0.001
Pandemic		1.7%	14.6%	34.3%	25.1%	12.1%	12.2%		<0.001
		**Physically Active Days per Week**							
	**0 days**	**1 day**	**2 days**	**3 days**	**4 days**	**5 days**	**6 days**	**7 days**	
Pre-pandemic	8.4%	17.3%	21.8%	20.1%	12.1%	8.1%	5.4%	6.7%	<0.001
Pandemic	14.7%	15.6%	17.7%	16.2%	11.1%	9.5%	5.7%	9.4%	<0.001

Pre-pandemic data were derived from the Health Behaviour in School-aged Children (HBSC) study conducted in 2018 [17].

## Data Availability

The raw data supporting the conclusion of this article will be made available by the authors upon reasonable request.
